# Safety, feasibility, and short-term-outcome of anal endoscopic submucosal dissection for anal intraepithelial neoplasia: an option for focal lesions?

**DOI:** 10.1007/s10151-023-02896-x

**Published:** 2023-12-15

**Authors:** F. Singhartinger, A. Gantschnigg, J. Holzinger, A. Wagner, J. Singhartinger, O. Koch, K. Emmanuel, J. Presl

**Affiliations:** 1 Department for Surgery, University Hospital Salzburg, Salzburg, Austria; 2 Department for Internal Medicine 1, University Hospital Salzburg, Salzburg, Austria; 3 Department for Gynecology and Obstetrics, Hospital Traunstein, Traunstein, Germany

**Keywords:** Endoscopic submucosal dissection, Anal intraepithelial neoplasia, Anal cancer

## Abstract

**Background:**

Anal intraepithelial neoplasia (AIN) appears in three different stages. AIN 1 and AIN 2 (p16 negative) are defined as low risk and unlikely to progress to invasive anal cancer. AIN 2 (p16 positive) and AIN 3 are of high risk and should be treated because progression rates to anal cancer are around 10% and treatment significantly reduces this risk. The correct treatment is still a matter of debate. Human papilloma virus (HPV) plays a role in the development of AIN. Our aim was to assess anal endoscopic dissection (aESD) as an intervention for AIN3.

**Methods:**

We retrospectively evaluated patients who underwent aESD for AIN 3 between December 2017 and March 2023. The interventional technique itself (duration, complications, size of specimen) and patient outcomes (recurrence, progression to anal cancer, re-intervention) were analyzed.

**Results:**

Fifteen patients with a median age of 52 years (23−78) underwent aESD for AIN 3. All tested specimens were positive for HPV. Median duration of intervention was 56.1 min, one delayed postinterventional bleeding occurred, and specimen size was 12.05 cm^2^. Median follow-up was 11.17 months. Three recurrences (20%) appeared: one was resected via biopsy and two were again treated with aESD. There was no progression to invasive anal cancer in the follow-up period.

**Conclusions:**

Anal endoscopic submucosal dissection seems to be a safe and feasible treatment for AIN. Recurrences are seldom and can be treated again with the same method. Nevertheless, indications for resection in comparison to radiofrequency ablation, pharmacological therapy, and watch-and-wait strategy are still unclear.

**Trial registration:**

Ethics commission of Salzburg, Austria, EK-Nr. 1056/2023. Keywords: Endoscopic submucosal dissection, anal intraepithelial neoplasia, anal cancer

**Supplementary Information:**

The online version contains supplementary material available at 10.1007/s10151-023-02896-x.

## Introduction

In 1976, Harald zur Hausen published his hypothesis about the connection between condylomata accuminata and cervical cancer [1]. Since then, this groundbreaking understanding of risk and pathogenesis of virally induced malignant diseases has increased in importance and led to a gain in knowledge [2]. Nowadays, the sexually transmitted infectious diseases human immunodeficiency virus (HIV) and human papilloma virus (HPV), which increase the risk of development of anal intraepithelial neoplasia (AIN), are regarded as an oncogenic pathway in the development of invasive anal squamous cell cancer (ASCC) [3, 4].

Studies have shown that men who have sex with men (MSM) and infection with HIV are risk factors for an infection with HPV [4–7]. HPV again promotes the development of AIN. This can be regarded as an important precursor lesion for ASCC [3, 8]. About 90% of ASCC are associated with HPV infection, the leading subtypes of which are HPV16 and 18 [9].

AIN is graded in three different types. AIN 1 and AIN 2 (p16 negative) is a benign neoplasia with low grade dysplasia, and is equivalent to low grade squamous intraepithelial lesion (LSIL). AIN 2 (p16 positive) and AIN 3 are considered to have a higher potential of malignant transformation due to high grade dysplasia, and is equivalent to high grade squamous intraepithelial lesion (HSIL) [10]. The specific factors that differentiate between AIN that is likely to regress and AIN that is likely to progress to anal cancer are still under debate and a topic of current research [11]. Studies suggest a rate of progression from AIN 3 to ASCC of 9–13% within 5 years [12].

The need for proper screening programs seems obvious because the development of AIN to ASCC is well known. However, to date no standardized guidelines on screening for AIN or ASCC exist. Screening strategies for AIN include anal cytology (AC) and high resolution anoscopy (HRA) [3]. AC is known to generate a high number of false negatives results whereas HRA is more sensitive but availability is limited. In our department, screening HRA is carried out in patients with acquired immunodeficiency syndrome or known history of AIN every 1–3 years but compliance is low.

Treatment of AIN is controversial. As mentioned above, no clear international guideline on screening and treatment of AIN is established. Treatment options range from watch-and-wait, local radiofrequency ablation, electrocautery ablation, medical ablation (imiquimod), or cryoablation up to radical surgical resection [13]. According to data from a recent trial by Palefsky et al., office-based electrocautery ablation is the most common treatment [14]. A comparative phase 3 clinical trial with 4459 patients recently showed a significant reduction in the risk for anal cancer in patients with AIN 3 who were treated (with local ablation or surgery) in comparison to active surveillance [14].

Anal endoscopic submucosal dissection (aESD) for AIN and early-stage ASCC has been reported in several case reports and series [15–18]. We present our experience on the safety, feasibility, and short-term outcome of our cohort with 15 patients with AIN treated with aESD.

## Methods

### Patient cohort

All patients treated with aESD for AIN in our tertiary referral center for interventional endoscopy, which performs around 10,000 investigations and interventions per year, were included in this study. The study period was from December 2017 to March 2023. Data on sex, age, underlying disease (immunodeficiency syndrome or not, HPV infection or not), stage of AIN (1–3), duration of intervention, size of specimen, resection status (R0, R1, Rx), follow-up, and recurrence were extracted from our prospectively maintained database. Agreement from the ethics committee responsible for our institution was obtained (ethics committee of Salzburg, EK-Nr. 1056/2023).

### Statistical analysis

Because the number of cases was small and there was no comparison to another group, only descriptive statistical analysis was carried out. Data was analyzed using mean values and standard distribution.

### High resolution anoscopy (HRA)

HRA is a screening tool for patients with increased risk for anal neoplasms [19]. The patient is usually placed in lateral left position (fetal position). A high resolution anoscope with up to ×40 magnification is used to examine the anal canal in a systematic manner and suspicious lesions should be biopsied. The visualization can be enhanced by application of acetic acid or Lugol’s iodine solution. In our department we use a modified technique with gastroscopes (Olympus GIF H190, Olympus, Tokyo, Japan) with an attached distant cap. The advantage is to also be able to examine the inner anal canal in retroflection. The disadvantage of this technique is the difficulty in visualizing the perianus/outer anal skin. Acetic acid and Lugol’s iodine solution are used as mentioned above. Any suspicious lesion for AIN or ASCC are biopsied. These examinations in our department are done by three experienced endoscopists who perform at least 1500 endoscopic procedures per year.

### Interventional technique

**Fig. 1:**
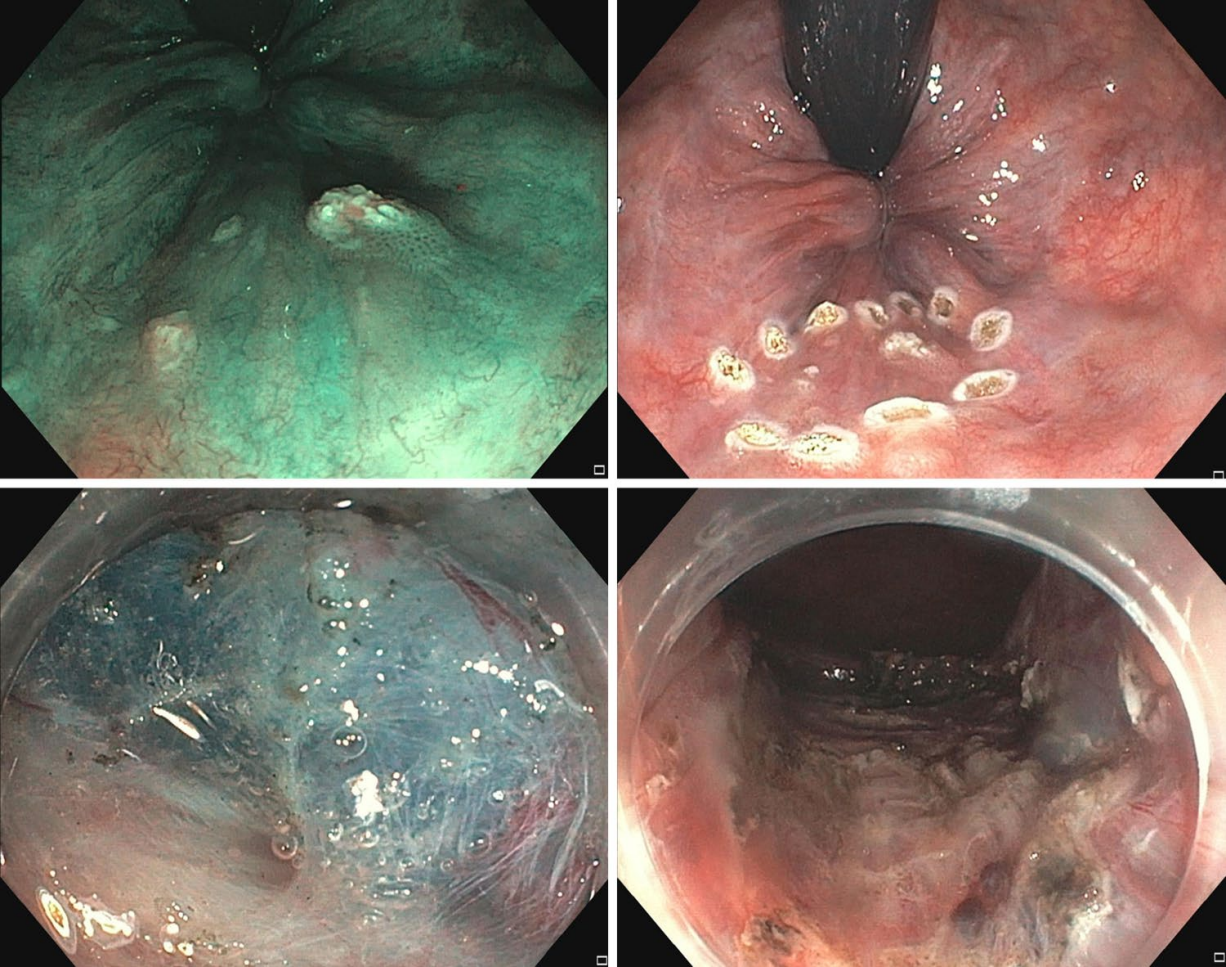
Top left: macroscopic narrow band imaging (NBI)-mode view of multifocal AIN 3; top right: lesion marked with argon plasma coagulation; bottom left: resection plane during ESD; bottom right: after resection

**Fig. 2:**
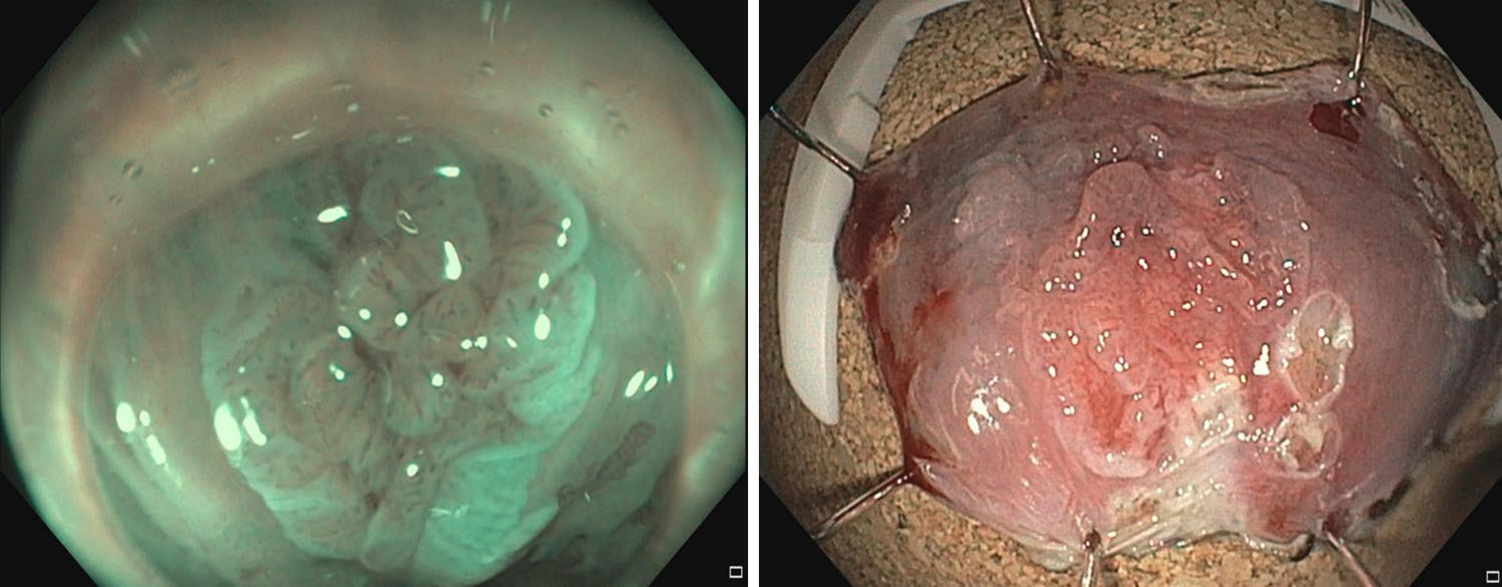
Left: AIN 3 in NBI mode; right: resected specimen

Three interventional endoscopy experts did all aESDs. Bowel preparation was similar to routine colonoscopy with 1 L of a macrogol-based solution. Interventions were carried out under continuous sedation with intravenously administered propofol or a combination of propofol and midazolam. The preferred endoscope was a hyperflexible pediatric coloscope (Olympus PCF H190TI, Olympus, Tokyo, Japan) to enable dissection in inversion as well as in antegrade position. After the resection margins were marked with argon plasma coagulation (APC), a lifting agent was injected in the submucosal space and aESD was done using a standard cutting device (DualKnife J or HookKnife, Olympus, Tokyo, Japan). No clip or suture closure of the wound was done (Fig. 1 and 2). Postinterventional pain was treated with oral non-steroidal antiphlogistics (NSAR) or metamizole, if necessary. Patients were usually discharged 1 day after the intervention. During follow-up, HRA biopsies were only taken when recurrence was suspected. HPV testing in follow-up examinations was only done when biopsies were taken, no cytology was carried out.

## Results

**Fig. 3:**
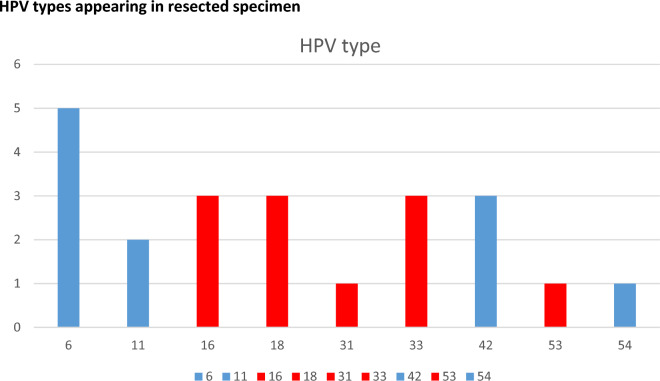
Distribution of HPV type in resected 13 specimens with proof of HPV. Red: high risk HPV; blue: low risk HPV

In the study period 15 patients (9 female, 6 male, median age 51.7 years (23–78)) underwent aESD for AIN. Demographic data and detailed information on surgery and follow-up is provided in Table 1. Six out of 15 patients were known to be HIV positive, and all HIV-positive patients were under antiretroviral therapy (ART). Preoperative biopsy showed AIN in every patient (AIN 1, *n* = 3; AIN 2, *n* = 1; AIN 3, *n* = 11). HPV was present in 13 out of 15 specimens; in two specimens no test on HPV was carried out (Fig. 3). Four patients had only high risk HPV strains, six patients had high risk and low risk HPV strains, and three patients had only low risk HPV strains. The R0 resection rate was 80% for high grade lesions (AIN 2 or 3) and 73% overall. This is because one patient was R0 for AIN 2 but with positive resection margins for AIN 1. Median operating time was 56.1 min (15–165 min) from first cut into the mucosa until completion of the resection with retrieval of the resected specimen. The extent of resection depended on the extent of the lesion plus 5 mm safety resection margin which was up to two-thirds of the anal circumference. Nevertheless no postoperative anal stenosis occurred. Furthermore no major complications occurred. Only one minor complication occurred (Clavien–Dindo grade 3a). The patient presented with delayed postoperative bleeding on postoperative day 15 which was managed by endoscopic clipping under sedation with propofol. No further intervention was necessary. Twelve of 15 (80%) participating patients completed at least one follow-up HRA; three have not had a follow-up yet because aESD was done in 2022 and first follow-up is still pending. Median follow-up in these 12 patients was 11.17 months (3−23). Median time to first follow-up was 5.17 (3–10) months. A second follow-up was only completed by 7 out of 15 (46.7%) patients after median 12.6 (6–18) months. Biopsies were only taken if a recurrent lesion was visible. Recurrence appeared in 3 out of 15 (20%) patients. Two patients were again treated with aESD; in one patient no lesion was detected after positive biopsy and so it was considered as resected by biopsy. R1 resection was not associated with recurrence.

**Table 1:** Demographic data and patients’ operative and postoperative course

Patients	*N* = 15
Sex	Female: *n* = 9; male: *n* = 6
Age, years	51.7 (23–78)
AIN in preoperative biopsy,* n* (%)	15/15 (100)
Histology of resected specimen	AIN 3, *n* = 11 (73.3%); AIN 2, *n* = 3 (27.3%); AIN 1, *n* = 1 (6.7%)
R0 resection (for AIN 2/3),* n* (%)	12/15 (80)
Size of specimen (cm^2^)	12.05 (2.25–36.1)
Duration of intervention (mins)	56.1 (15–165)
Minor complication,* n* (%)	1/15 (delayed bleeding) (6.7)
Major complication,* n* (%)	0/15 (0)
First follow-up,* n* (%)	12/15 (80)
Time to first follow-up, months	5.17 (3–10)
Second follow-up,* n* (%)	7/15 (46.7)
Time to second follow-up, months	12.6 (6–18)
Third follow-up,* n* (%)	3/15 (20)
Time to third follow-up, months	16.7 (9–23)
Fourth follow-up,* n* (%)	1/15 (7.7)
Time to third follow-up, months	12

## Discussion

ESD is a well-established technique used in several regions of the gastrointestinal tract. However, because of its technical difficulty ESD in the anal canal is really pushing the limits of endoscopy.

This is, to our knowledge, the biggest cohort of patients with anal neoplasms treated with ESD in a single institution. Case reports and series have shown the possibility of ESD in the anal canal, but larger cohorts or prospective studies are lacking [14–17].

As mentioned above, international screening programs or guidelines for anal HPV infections and AIN are rare, although some national recommendations exist [20]. Even the strategies of screening (anal cytology for high risk HPV versus digital rectal examination with visual examination of the outer anal canal) are still a matter of debate, as is the cohort that is needed to be screened for AIN (HIV-positive patients, MSM patients) [21, 22]. HIV-positive patients which are under frequent surveillance during ART in the infectiology department at our university hospital are referred to endoscopic surveillance every 1–3 years. As mentioned above, compliance is low. This might be due to the young age of the patients and their lack of awareness about precancerous anal lesions. Furthermore, AIN is, in most cases, an asymptomatic disease.

HPV was present in all specimens that have been tested for HPV. This is well in line with previous data on the high prevalence of HPV positivity in AIN [5]. However, less than 50% of our cohort was positive for the predominant high risk HPV strains (16 and 18) in ASCC as described by Saraiya et al. [9], but still 77% were positive for high risk HPV strains (Fig. 3). Of course, this has limited relevance because of the low case number in our investigation.

Treatment options for AIN vary. A review by Brogden et al. in 2021 stated a wide variety of therapeutic options [13]. These include non-invasive options like topical imiquimod (5%) and topical 5-fluorouracil as well as invasive techniques like electrocautery ablation and surgical excision. Endoscopic resection was not mentioned. According to the authors there is no evident superiority for either of the aforementioned therapies. The phase 3 clinical trial by Palefsky et al. from 2022 showed a reduction in the risk for anal cancer in patients treated in comparison to watch-and-wait [14]. Although this study included an impressive number of patients (*n* = 4459), many different treatment strategies were applied and not compared individually. The most frequently used intervention was office-based electrocautery ablation. This is, by far, the highest quality evidence of treatment of AIN to date. Although our data cannot be compared with a randomized controlled trial because of its retrospective character, low case number, and short follow-up, our patient cohort experienced no progression to ASCC.

According to current literature a clear recommendation for one or another treatment option cannot be made. Surgical excision, infrared coagulation, and electrocautery ablation show quite high recurrence rates, up to 79% [23–26]. In a prospective study by Chang et al., 79.3% of HIV-positive patients that were treated with surgical excision developed recurrence with a mean time to recurrence of 12 months [23]. Regarding topical non-invasive treatment, an investigation by Cranston et al. reported a local recurrence rate of 20.8%, and 32.1% had a new lesion at another location [27].

Regarding recurrence rates, aESD might be superior to other invasive treatment techniques. This might be due to the superior visibility of anal lesions of the inner anal canal with high resolution endoscopes and applied filters and thereby increased R0 resection rates. Of course, validity is limited because of the short follow-up period.

Patients usually were discharged from the hospital 1 day after the intervention and analgesic therapy was done mostly with only oral non-steroidal anti-inflammatory medication. This is why in the future aESD could be planned in an outpatient setting. Nevertheless aESD in daily practice should be limited to patients with small lesions, recurrence, or high risk features because it is more invasive than ablation or topical therapy. Furthermore it should be carried out in centers with adequate expertise.

Limitations of the study are the low case number and short follow-up period, as mentioned above. Furthermore no control group (i.e., versus surgical resection) is presented because all patients with AIN in the inner anal canal in our department are treated with aESD.

Overall this investigation shows the feasibility and safety of aESD for AIN. Furthermore, aESD might be a treatment option associated with low recurrence rates and a high percentage of R0 resections. We hope to thereby contribute to a better understanding of treatment strategies for AIN, although this investigation ought to be interpreted as preliminary.

## Conclusion

Further studies investigating the reasons for the heterogeneous progression risk of AIN to ASCC need to be done. Then, in addition, prospective trials comparing the different treatment options of AIN are necessary.

## Supplementary Information

Below is the link to the electronic supplementary material.

Supplementary file1 (DOCX 15 KB)

## Data Availability

All data generated or analyzed during this study are included in this published article and its supplementary information files.
