# Supplementary material for: Safety, feasibility, and short-term-outcome of anal endoscopic submucosal dissection for anal intraepithelial neoplasia: an option for focal lesions?
Source: Tech Coloproctol. 2023 Dec 15;28(1):18. doi: 10.1007/s10151-023-02896-x (PMC10724311; doi:10.1007/s10151-023-02896-x)
Supplement: Supplementary file 1 — Supplementary file1 (DOCX 15 KB) [file 10151_2023_2896_MOESM1_ESM.docx]

**Supplementary electronic data: Detailed data on all 15 patients**

| Patient | Age | Sex | HIV | Histology in biopsy | Size of resected specimen (mmxmm) | Duration of intervention (min) | Definite histology | Type of HPV | Resection status | 1. follow-up (months) | 2. follow-up (months) | 3. follow-up (months) | 4. follow-up (months) | Complication |
| --- | --- | --- | --- | --- | --- | --- | --- | --- | --- | --- | --- | --- | --- | --- |
| 1 | 78 | Female | - | AIN 1 | 42x36 | 40 | AIN 3 | high risk | R0 | 10 |  |  |  | None |
| 2 | 43 | Male | - | AIN 3 | 60x25 | 75 | AIN 3 | 16/6/11 | R0 | 3 |  |  |  | None |
| 3 | 78 | Female | - | AIN 1 | 85x24 | 70 | AIN 3 | negative | R1 | 6 | 18 |  |  | None |
| 4 | 54 | Male | + | AIN 2 | 50x22 | 55 | AIN 2 | 33/11 | R0 | 3 | 10 | 18 |  | None |
| 5 | 27 | Male | + | AIN 3 | n.a. | 25 | AIN 3 | 6 | Rx | 5 |  |  |  | None |
| 6 | 26 | Male | + | AIN 1 | 60x30 | 95 | AIN 3 | 42 | R0 | 8 |  |  |  | None |
| 7 | 23 | Female | + | AIN 3 | 33x18 | 15 | AIN 3 | HPV 6/18 | R0 | 6 | 12 | 23 |  | None |
| 8 | 27 | Male | + | AIN 3 | 25x15 | 50 | AIN 2 | 18/42 | R0 (AIN 2); R1 (AIN 1) | 6 |  |  |  | None |
| 9 | 78 | Female | - | AIN 3 | 40x38 | 34 | AIN 3 | 16 | R0 | 3 | 15 |  |  | None |
| 10 | 53 | Female | - | AIN 3 | 95x38 | 85 | AIN 3 | 16 | R0 | 3 | 15 |  |  | None |
| 11 | 68 | Female | - | AIN 3 | 25x20 | 20 | AIN 2 | 53/42 | R0 | 6 | 12 |  |  | None |
| 12 | 70 | Female | - | AIN 3 | 18x18 | 62 | AIN 3/pTis | 33 | R0 | 3 | 6 | 9 | 12 | None |
| 13 | 39 | Female | - | AIN 3 | 15x15 | 165 | AIN 1 | 6/54 | R1 | 10 |  |  |  | None |
| 14 | 55 | Female | - | AIN 3 | 33x26 | 33 | AIN 3 | negative | R0 | 3 |  |  |  | None |
| 15 | 56 | Male | + | AIN 3 | 38x24 | 17 | AIN 3 | 6/18/31/33 | R0 | 6 | 18 |  |  | Bleeding 15 days after intervention |
